# Adaptive optics scanning laser ophthalmoscopy in a heterogenous cohort with Stargardt disease

**DOI:** 10.1038/s41598-024-74088-y

**Published:** 2024-10-09

**Authors:** Mital Shah, Susan M. Downes, Hannah E. Smithson, Laura K. Young

**Affiliations:** 1 Oxford Eye Hospital, John Radcliffe Hospital, Oxford University Hospitals NHS Foundation Trust, Oxford, OX3 9DU UK; 2 Nuffield Laboratory of Ophthalmology, Nuffield Department of Clinical Neurosciences, Level 6 John Radcliffe Hospital, University of Oxford, Headley Way, Oxford, OX3 9DU UK; 3 Department of Experimental Psychology, University of Oxford, Oxford, OX2 6GG UK; 4 Biosciences Institute, Newcastle University, Newcastle, NE2 4HH UK

**Keywords:** Retinal imaging, Adaptive optics, Adaptive optics scanning laser ophthalmoscope, AOSLO, Stargardt disease, ABCA4, Hereditary eye disease, Medical imaging

## Abstract

Image based cell-specific biomarkers will play an important role in monitoring treatment outcomes of novel therapies in patients with Stargardt (STGD1) disease and may provide information on the exact mechanism of retinal degeneration. This study reports retinal image features from conventional clinical imaging and from corresponding high-resolution imaging with a confocal adaptive optics scanning laser ophthalmoscope (AOSLO) in a heterogenous cohort of patients with Stargardt (STGD1) disease. This is a prospective observational study in which 16 participants with clinically and molecularly confirmed STGD1, and 7 healthy controls underwent clinical assessment and confocal AOSLO imaging. Clinical assessment included short-wavelength and near-infrared fundus autofluorescence, spectral-domain optical coherence tomography, and macular microperimetry. AOSLO images were acquired over a range of retinal eccentricities (0°–20°) and mapped to areas of interest from the clinical images. A regular photoreceptor mosaic was identified in areas of normal or near normal retinal structure on clinical images. Where clinical imaging indicated areas of retinal degeneration, the photoreceptor mosaic was disorganised and lacked unambiguous cones. Discrete hyper-reflective foci were identified in 9 participants with STGD1 within areas of retinal degeneration. A continuous RPE cell mosaic at the fovea was identified in one participant with an optical gap phenotype. The clinical heterogeneity observed in STGD1 is reflected in the findings on confocal AOSLO imaging.

## Introduction

Stargardt (STGD1) disease is the commonest inherited macular dystrophy in adults and children^1^ and results in progressive loss of central vision. It has a reported annual incidence in the United Kingdom of between 0.110 and 0.128 per 100,000 individuals^2^. The genetic basis of this disease has been well documented and STGD1 is caused by variants in the *ABCA4* gene. STGD1 is one of the *ABCA4* retinopathies^3,4^. The *ABCA4* gene is associated with significant phenotypic and genetic heterogeneity with more than 900 *ABCA4* disease causing variants reported to date^5^.

There are a number of ongoing different treatment trials in patients with STGD1 disease comprising gene replacement therapy, cell-based therapy and pharmacotherapy^1,6^. With the development of these new therapies cell-specific biomarkers will play an important role in assessing and monitoring response to treatment outcomes.

Adaptive optics scanning laser ophthalmoscopy (AOSLO) enables in vivo imaging with resolution at the cellular level, thus making cell-specific imaging biomarkers possible. Of particular relevance in STGD1, is that individual photoreceptors, including foveal cones and peripheral rods, can be visualised in the living human eye. AOSLO imaging in STGD1 will provide information on the natural history of the disease at the level of the photoreceptors that will be useful for assessing the response to future treatments and may help to provide information on the exact mechanism of retinal degeneration. Understanding the significance of structural changes observed with in vivo AOSLO retinal imaging is a prerequisite to using these image-based biomarkers in clinical practice. Studies reporting the results of AOSLO in vivo imaging in patients with STGD1 and *ABCA4* retinopathies are crucial to improve understanding and interpretation of observed structural features in these heterogenous phenotypes. We report a cohort of participants with STGD1 disease at various degrees of disease progression using confocal AOSLO.

## Methods

Sixteen participants with a clinical diagnosis of STGD1 disease and at least one pathogenic variant in *ABCA4*, and 7 controls, underwent clinical assessment and confocal AOSLO imaging. All participants provided written informed consent prior to participation in this study and National Health Service Research Ethics Committee approval was obtained from the South Central—Oxford B Research Ethics Committee (reference—15/SC/0144) and the Yorkshire & The Humber—Sheffield Research Ethics Committee (reference—16/YH/0265). This study was conducted in adherence to the tenets of the Declaration of Helsinki^7^. Participants did not receive compensation or an incentive for participating in this study.

### Clinical assessment

Clinical assessment included best corrected visual acuity, slit lamp biomicroscopy, colour fundus photography (TRC-50DX Mydriatic Retinal Camera, Topcon), short- wavelength and near-infrared fundus autofluorescence imaging (Spectralis; Heidelberg Engineering), spectral-domain optical coherence tomography (SD-OCT) (Spectralis; Heidelberg Engineering), and macular microperimetry with a 10-2 testing grid and 4-2 projection strategy (Macular Integrity Assessment; CenterVue).

### Macular microperimetry

Macular microperimetry has the dual purpose of quantifying retinal sensitivity and measuring the preferred retinal locus (PRL). It was performed under mesopic light conditions to elicit both rod and cone responses, prior to pupil dilation, and after 20 min of dark-adaptation. All examinations were performed monocularly, with patching of the fellow eye. A 10-2 grid was used with a Goldmann III stimulus and a 4-2 projection strategy. The centre of all fixation points recorded after the initial 10 s of a macular microperimetry examination was used to determine the PRL on the retinal surface. A 63% bivariate contour ellipse (an ellipse that encompasses 63% of all fixation events^8^) was used as a measure of fixation stability. Average retinal sensitivity (referred to as retinal sensitivity), was determined by calculating the mean threshold of all points assessed in the 10-2 grid. PRL sensitivity was determined by calculating the average threshold of the four macular microperimetry test points centred on the PRL position.

### AOSLO imaging

Confocal near-infrared reflectance imaging (wavelength = 850 nm, full width at half maximum = 50 nm) was carried out using a previously described instrument^9^. A periscope is used with a beam splitter to enable the participant to be shown a distant visual display containing a fixation target during imaging. By using the periscope to raise the beam splitter above the main optical system, a wide field of view for distant fixation targets can be achieved. Participants were asked to wear a wax dental impression on an adjustable bite-bar to ensure that the head is kept still during the AOSLO imaging. Alignment of the eye to the system was performed using the wavefront-sensor image as a guide and the use of refractive correction (prescription glasses or trial lenses) was based on image quality. Images were acquired at a rate of 30 Hz with a 1.6° × 1.0° field of view, and retinal locations were targeted by directing the participant’s gaze using a fixation target on a display located 1.75 m from the eye. In participants with extra-foveal fixation, an adjustment was made to account for the position of the PRL relative to the foveal centre and retinal features from fundus images were used to check accuracy. Pharmacologic pupil dilation and cycloplegia was not performed routinely, which allowed participants to focus naturally on the display. The selection of a long imaging wavelength reduced illumination-related pupil constriction and, to maximise natural pupil dilation, all imaging was performed in a darkened room. Regions of interest were selected prior to imaging using multimodal retinal imaging collected during the clinical assessment, and during AOSLO image capture fundus images were used with a graphical user interface to target these regions. The regions of interest in participants with STGD1 were selected to include the leading disease edge, the transition from an area of relatively normal to degenerated retina and the site of active degeneration. Due to the variability in retinal phenotype and disease severity between participants, a standard set of retinal locations for imaging were not used. A search in the z-plane was carried out for each participant at the time of AOSLO imaging in order to optimise structures visible corresponding to the photoreceptor mosaic inner segment/outer segment junction. The identification of cone photoreceptors was based on the spatial layout and packing geometry of bright dots and their scale, following the search in the z-plane. Regions of unambiguous cones were identified in areas of retinal degeneration.

### AOSLO image scaling

The Gullstrand schematic human eye with an axial length of 24 mm yields a conversion factor of 291 μm per degree of visual angle^10^, which was scaled by the participant’s axial length measured with an IOLMaster (Carl Zeiss Meditec) to determine the scale of each retinal image in microns per pixel. Axial length was assumed to be constant with eccentricity as it varies by less than 2% across the range of retinal eccentricities imaged in this study (0°–20°)^11^. Raw frames were corrected for motion distortions before being averaged.

### Discrete hyper-reflective foci

Averaged AOSLO images were manually reviewed to identify discrete hyper-reflective foci. These hyper-reflective foci were noted to be distinct from bright photoreceptors that were identified in areas of normal or near normal retinal structure as they did not have a regular packing arrangement and were only identified in areas of retinal degeneration. The discrete hyper-reflective foci were characterised with a custom-made automated image processing pipeline that was developed and implemented in the Python programming language (Python Software Foundation. Python Language Reference, version 2.7. Available at http://www.python.org). Within a frame, 200 discrete hyper-reflective foci were identified with a local maximum function (the 200 brightest regions). These discrete hyper-reflective foci were cropped and aligned to create an image stack that was then averaged. The size of the average discrete hyper-reflective foci image was determined by fitting a Gaussian profile, and the size is reported as full width at half maximum. This process was repeated for each averaged AOSLO image containing discrete hyper-reflective foci.

### Statistical analyses

Statistical analyses were carried out using the statistical functions module of the open-source software SciPy (version 1.14, available at https://scipy.org/install/). Welch’s t-test, an independent samples test that does not assume equality of variances, was used to compare between participants with STGD1 and control participants for significant differences in PRL position, bivariate contour ellipse area (BCEA) and retinal sensitivity. A *p*-value of less than 5% was considered statistically significant. Bonferroni correction was used for multiple-comparison correction. A linear least-squares regression model was used to test the relationship between discrete hyper-reflective foci size and retinal eccentricity.

## Results

Sixteen participants (7 male, 9 female) from 16 families with Stargardt disease, and 7 controls (1 male, 6 female) underwent clinical assessment and confocal AOSLO imaging. AOSLO images near the foveal centre have been previously published for 5 control participants (C01, C02, C04, C05, C06)^9^. Demographics and baseline clinical characteristics of all participants are summarised in Table 1 and Table 2. Molecular genetic testing identified 36 variants in *ABCA4*. Variants in genes other than *ABCA4* were identified in one participant (S09) in whom a single variant in RP1 was identified (c.4603C > G p.(Pro1535Ala)).

**Table 1:** Demographics and baseline clinical characteristics of participants with Stargardt disease.

Participant No./Sex/Age	Age at symptom onset	LogMAR BCVA		Refraction						*ABCA4* genotype*	Retinal sensitivity (dB)		Axial length (mm)	
				OD			OS							
		OD	OS	SPH	CYL	AX	SPH	CYL	AX		OD	OS	OD	OS
S01/F/70’s	70’s	0.60	0.10	3.00	− 1.50	105	3.00	− 0.25	075	c.3004C > T p.(Arg1002Trp) and c.5714 + 5G > A	6.70	13.10	22.86	22.70
S02/M/30’s	20’s	0.40	0.30	− 0.25	− 0.50	168	∞	− 0.25	000	c.5461-10 T > C	21.50	23.30	24.53	24.41
S03/M/50’s	30’s	0.34	0.04	− 2.75	− 1.00	155	− 2.75	− 0.75	175	c.2588G > C (p.Gly863Ala) and c.4326C > A (p.Asn1442Lys)	11.00	13.60	25.45	25.51
S04/M/40’s	40’s	0.02	0.40	0.50	0.25	116	3.00	− 1.50	042	c.5461-10 T > C and c.6718A > G (p.Thr2240Ala)	26.40	24.60	23.30	22.66
S05/F/20’s	10’s	0.90	0.94	− 2.25	− 0.50	022	− 1.75	− 0.75	156	c.4537dupC (p.Gln1513fs), c.6391G > A (p.Glu2131Lys), c.1411G > A (p.Glu471Lys) and c.455G > A (p.Arg152Gln)	16.60	18.10	23.68	23.49
S06/F/20’s	20’s	0.12	0.66	− 0.50	− 0.25	010	− 0.75	− 0.50	020	c.3259G > A p.(Glu1087Lys) and c.6089G > A p.(Arg2030Gln)	23.70	21.20	22.69	22.66
S07/M/20’s	10’s	0.80	0.80	− 3.07	− 0.08	090	− 3.09	− 0.11	090	c.1230A > G (p.Ile410Met), c.4216C > T (p.His1406Tyr) and c.6148G > C (p.Val2050Leu)	20.90	21.20	25.54	25.58
S08/M/20’s	10’s	0.96	0.94	− 3.32	− 1.09	033	− 3.03	− 1.95	153	c.2588G > C (p.Gly863Ala), c.1715G > A (p.Arg572Gln) and c.5461-10 T > C	16.20	15.80	25.26	25.17
S09/F/30’s	30’s	0.52	0.08	− 2.25	− 0.50	085	− 2.25	–	–	c.5882G > A p.(Gly1961Glu), c.3259G > A p.(Glu1087Lys) and c.5693G > A p.(Arg1898His)	23.80	23.40	24.45	24.29
S10/F/30’s	< 10	1.04	0.96	1.25	–	–	2.00	− 0.50	114	c.5018 + 2 T > C and c.6316C > T p.(Arg2106Cys)	18.60	20.50	22.15	21.89
S11/F/10’s	< 10	0.90	0.96	− 1.00	− 2.00	180	− 1.00	− 2.00	180	c.3064G > A p.(Glu1022Lys) and c.6709A > C p.(Thr2237Pro)	11.10	–	23.33	23.16
S12/M/20’s	10’s	1.02	0.98	0.25	− 1.00	002	1.50	− 1.00	178	c.3085C > T p.(Gln1029*) and c.5882G > A p.(Gly1961Glu)	19.30	29.70	22.61	22.13
S13/F/40’s	20’s	1.08	0.94	− 2.25	− 0.75	021	− 2.25	− 0.75	138	c.2588G > C p.(Gly863Ala) and c.1A > G p.(Met1Val)	19.60	18.40	23.74	23.58
S14/F/40’s	30’s	0.96	1.08	− 1.00	− 0.75	041	− 1.25	− 1.75	011	c.6089G > A p.(Arg2030Gln) and c.5461-10 T > C	12.20	12.00	23.12	23.31
S15/F/40’s	10’s	0.90	0.96	− 0.50	–	–	− 1.00	–	–	c.634C > T (p.Arg212Cys) and c.4457C > T (p.Pro1486Leu)	14.20	14.50	22.96	22.95
S16/M/30’s	20’s	1.00	0.94	− 2.25	− 0.50	009	− 1.25	− 0.75	007	c.6079C > T (p.Leu2027Phe) and c.3322C > T (p.Arg1108Cys)	16.60	13.80	23.99	24.08

*All variants are heterozygous, *AX* axis, *BCVA* best corrected visual acuity, *CYL* cylinder, *LogMAR* Logarithm of the Minimum Angle of Resolution, *OD* right eye, *OS* left eye, *SPH* sphere. The median retinal sensitivity for all participants was 18.4 dB (IQR 7.35 dB, range 6.7–29.7 dB).

**Table 2:** Demographics and baseline clinical characteristics of control participants.

Participant No./Sex/Age	LogMAR BCVA		Refraction						PRL distance from fovea (°)		63% BCEA (deg^2^)		Retinal sensitivity (dB)		Average PRL sensitivity [SD] (dB)		Axial length (mm)	
			OD			OS												
	OD	OS	SPH	CYL	AX	SPH	CYL	AX	OD	OS	OD	OS	OD	OS	OD	OS	OD	OS
C01/F/30’s	− 0.22	− 0.16	–	–	–	–	–	–	0.77	0.35	0.1	0.1	28.0	28.7	30.0 [1.4]	31.0 [3.0]	22.0	21.9
C02/F/20’s	0.00	0.00	–	–	–	–	–	–	0.59	0.45	0.2	0.2	28.9	29.0	31.3 [1.8]	31.0 [1.4]	22.4	22.6
C03/F/20’s	− 0.36	− 0.36	− 1.50	− 0.75	148	− 1.75	− 0.25	179	0.49	0.33	0.3	0.3	26.8	27.0	29.3 [1.1]	29.0 [1.4]	24.4	24.3
C04/F/30’s	− 0.12	− 0.10	− 3.00	–	–	− 3.00	–	–	0.44	0.32	0.5	0.3	25.7	27.3	26.5 [1.7]	30.5 [0.9]	25.2	25.2
C05/F/40’s	− 0.10	0.02	− 2.25	–	–	− 0.25	–	–	0.05	0.16	0.4	0.6	26.6	27.5	28.5 [1.7]	27.0 [0]	24.0	23.3
C06/M/40’s	− 0.32	− 0.32	0.50	− 0.25	090	0.25	–	–	0.17	0.51	0.2	0.1	26.8	26.6	29.0 [1.0]	28.5 [0.9]	23.7	23.9
C07/F/30’s	− 0.20	− 0.22	∞	0.50	075	∞	0.25	088	0.53	0.47	2.2	1.2	26.2	27.3	28.5 [0.9]	29.5 [0.9]	22.0	22.0

*AX* axis, *BCEA* bivariate contour ellipse area, *BCVA* best corrected visual acuity, *CYL* cylinder, *LogMAR* Logarithm of the Minimum Angle of Resolution, *OD* right eye, *OS* left eye, *PRL* preferred retinal locus, *SPH* sphere. The median PRL eccentricity was 0.45° from the foveal centre (IQR 0.18°, range 0.05°–0.99°), median 63% BCEA was 0.3 deg^2^ (IQR 0.3 deg^2^, range 0.1 deg^2^–2.2 deg^2^), the median retinal sensitivity was 27.2 dB (IQR 1.2 dB, range 25.7–29.0 dB), and the median PRL sensitivity was 29.2 dB (IQR 1.9 dB, range 26.5–31.3 dB).

### Clinical assessment

The retinal phenotypes of participants with Stargardt disease are illustrated in Supplementary Fig. 1 and summarised in Table 3.

**Table 3:** Retinal phenotype in participants with Stargardt disease.

Participant	Colour	SW-FAF	NI-FAF	SD-OCT
S01	OU: central macular and peripapillary chorioretinal atrophy	OU: reticular pattern of AF abnormalities extending external to the arcades, peripapillary hypo-AF	N/A	OU: common phenotype of SD-OCT abnormalities. OS: foveal sparing
S02	OU: irregular central macular RPE reflex, retinal flecks	OU: reticular pattern of AF abnormalities extending external to the arcades	N/A	OU: common phenotype of SD-OCT abnormalities
S03	OU: chorioretinal atrophy within the vascular arcades and central macula	OU: reticular pattern of AF abnormalities extending external to the arcades	N/A	OU: common phenotype of SD-OCT abnormalities, foveal sparing
S04	OU: retinal flecks extending external to the arcades	OU: common phenotype of AF abnormalities, hyper-AF retinal flecks	OU: common phenotype of AF abnormalities	OU: common phenotype of SD-OCT abnormalities, foveal sparing
S05	OU: central macular chorioretinal atrophy	OU: reticular pattern of AF abnormalities extending external to the arcades	N/A	OU: common phenotype of SD-OCT abnormalities, early features of ORD
S06	OU: irregular central macular RPE reflex	OU: common phenotype of AF abnormalities	OU: common phenotype of AF abnormalities	OU: common phenotype of SD-OCT abnormalities, hyper-reflective foci predominantly within the ONL, early features of ORD
S07	OU: central macular irregular RPE reflex, retinal flecks	OU: common phenotype of AF abnormalities	N/A	OU: common phenotype of SD-OCT abnormalities, early features of ORD
S08	OU: central macular chorioretinal atrophy	OU: reticular pattern of AF abnormalities extending external to the arcades	N/A	OU: common phenotype of SD-OCT abnormalities, early features of ORD
S09	OU: irregular central macular RPE reflex	OU: common phenotype of AF abnormalities	N/A	OD: common phenotype of SD-OCT abnormalities, early features of ORD. OS: optical gap phenotype
S10	OU: central macular irregular RPE reflex, retinal flecks	OU: common phenotype of AF abnormalities, hyper-AF retinal flecks	OU: common phenotype of AF abnormalities	OU: common phenotype of SD-OCT abnormalities, hyper-reflective foci predominantly within the ONL, early features of ORD
S11	OU: central macular chorioretinal atrophy	OU: reticular pattern of AF abnormalities extending external to the arcades	OU: common phenotype of AF abnormalities	OU: common phenotype of SD-OCT abnormalities
S12	OU: irregular central macular RPE reflex	OU: common phenotype of AF abnormalities	OU: common phenotype of AF abnormalities	OU: common phenotype of SD-OCT abnormalities, hyper-reflective foci predominantly within the ONL, early features of ORD
S13	OU: central macular chorioretinal atrophy, retinal flecks	OU: common phenotype of AF abnormalities, hyper-AF retinal flecks	OU: common phenotype of AF abnormalities	OU: common phenotype of SD-OCT abnormalities. OD: epiretinal membrane
S14	OU: central macular chorioretinal atrophy, retinal flecks	OU: common phenotype of AF abnormalities, hyper-AF retinal flecks	OU: common phenotype of AF abnormalities	OU: common phenotype of SD-OCT abnormalities
S15	OU: central macular chorioretinal atrophy, retinal flecks	OU: common phenotype of AF abnormalities, hyper-AF retinal flecks	OU: common phenotype of AF abnormalities	OU: common phenotype of SD-OCT abnormalities, early features of ORD
S16	OU: central macular chorioretinal atrophy, retinal flecks	OU: common phenotype of AF abnormalities, hyper-AF retinal flecks	OU: common phenotype of AF abnormalities	OU: common phenotype of SD-OCT abnormalities

*AF* autofluorescence, *NI-FAF* near-infrared fundus autofluorescence, *OD* right eye, *ONL* outer nuclear layer, *ORD* outer retinal degeneration, *OS* left eye, *OU* both eyes, *RPE* retinal pigment epithelium, *SD-OCT* spectral-domain optical coherence tomography, *SW-FAF* short-wavelength fundus autofluorescence.

All participants showed a common phenotype of short-wavelength fundus autofluorescence (SW-FAF) abnormalities with central macular hypo-autofluorescence surrounded by a ring of hyper-autofluorescence. Six participants showed additional SW-FAF abnormalities with a reticular pattern consisting of hyper-autofluorescence flecks and areas of hypo-autofluorescence, which ranged in extent from within the central macula to external to the vascular arcades. All participants except one (S01) exhibited peripapillary sparing of SW-FAF abnormalities.

Nine participants underwent near-infrared fundus autofluorescence (NI-FAF) imaging and the observed pattern of autofluorescence abnormalities were similar to those seen with SW-FAF, but were greater in extent over the same retinal area.

A common phenotype on SD-OCT of retinal thinning with loss of outer retinal layers, with or without loss of the inner retinal layers, was observed in all participants with Stargardt disease. Three participants (S01, S03 and S04) exhibited foveal sparing in one or both eyes. One participant (S09) exhibited an optical gap phenotype in their left eye with loss of the ellipsoid zone and interdigitation zone and preservation of the underlying RPE.

### Preferred retinal locus

Details of the PRL eccentricity and sensitivity of all participants are summarised in Table 2 and Table 4. PRL position, bivariate contour ellipse area, retinal sensitivity and PRL sensitivity differed significantly (*p* < 0.001) between the STGD1 and control participant groups. An adjusted *p*-value of less than 1.25%, after Bonferroni correction, was considered to be statistically significant.

**Table 4:** Retinal structure and function at the preferred retinal locus in participants with Stargardt disease.

Participant	Eye	PRL distance from fovea (**°**)	Average PRL sensitivity [SD] (dB)	63% BCEA (deg^2^)	SW-FAF	NI-FAF	SD-OCT		
							ELM	EZ	RPE/Bruch’s
S01	OD	3.11	1.5 [2.6]	7.00	Hypo-AF	N/A	Absent	Absent	Absent
	OS	0.01	17.0 [2.2]	1.20	Hypo-AF	N/A	Present	Present	Present
S02	OD	0.04	2.0 [3.5]	0.30	Hypo-AF	N/A	Absent	Absent	Present
	OS	0.03	1.0 [1.7]	0.20	Hypo-AF	N/A	Absent	Absent	Present
S03	OD	0.04	0.3 [0.4]	1.60	Hypo-AF	N/A	Absent	Absent	Present
	OS	0.03	5.3 [6.9]	1.30	Normal	N/A	Present	Present	Present
S04	OD	0.03	26.0 [0]	0.50	Normal	Hypo-AF	Present	Present	Present
	OS	0.09	24.5 [6.1]	0.60	Normal	Hypo-AF	Present	Present	Present
S05	OD	4.42	21.8 [2.4]	0.80	Hypo-AF	N/A	Absent	Absent	Present
	OS	1.97	10.5 [10.5]	3.60	Hypo-AF	N/A	Absent	Absent	Present
S06	OD	0.02	3.8 [6.5]	0.70	Hypo-AF	Hypo-AF	Absent	Absent	Present
	OS	1.91	13.5 [13.5]	1.40	Hypo-AF	Hypo-AF	Present	Present	Present
S07	OD	4.13	23.0 [1.4]	4.70	Normal	N/A	Present	Present	Present
	OS	2.94	21.0 [2.8]	3.10	Hypo-AF	N/A	Present	Present	Present
S08	OD	8.06	20.0 [2.2]	5.50	Hypo-AF	N/A	Present	Present	Present
	OS	8.66	20.5 [3.0]	5.70	Hypo-AF	N/A	N/A	N/A	N/A
S09	OD	2.56	20.0 [7.1]	1.80	Hypo-AF	N/A	Present	Present	Present
	OS	0.30	16.0 [4.2]	1.20	Hypo-AF	N/A	Present	Absent	Present
S10	OD	5.30	19.0 [1.0]	4.30	Normal	Normal	Present	Present	Present
	OS	5.90	19.5 [2.6]	3.80	Normal	Normal	Present	Present	Present
S11	OD	21.35	13.0 [3.7]	14.90	Hypo-AF	Hypo-AF	Present	Absent	Present
	OS	16.81	N/A	6.40	Hypo-AF	Hypo-AF	Present	Absent	Present
S12	OD	5.53	17.3 [10.1]	4.50	Normal	Normal	Present	Present	Present
	OS	5.96	31.5 [4.5]	4.90	Normal	Normal	Present	Present	Present
S13	OD	10.30	22.0 [3.7]	7.60	Normal	Normal	Present	Present	Present
	OS	9.64	23.5 [1.7]	6.20	Hyper-AF	Hyper-AF	Present	Present	Present
S14	OD	4.79	0.5 [0.9]	7.60	Hypo-AF	Hypo-AF	Absent	Absent	Present
	OS	6.62	14.0 [5.1]	3.10	Hypo-AF	Hypo-AF	Absent	Absent	Present
S15	OD	9.06	15.0 [7.1]	1.50	Normal	Normal	Present	Present	Present
	OS	8.26	18.5 [4.3]	3.40	Hypo-AF	Hypo-AF	Present	Present	Present
S16	OD	10.12	17.0 [1.0]	10.00	Hypo-AF	Hypo-AF	Present	Present	Present
	OS	8.36	16.5 [0.9]	12.20	Hyper-AF	Hypo-AF	N/A	N/A	N/A

*AF* autofluorescence, *ELM* external limiting membrane, *EZ* ellipsoid zone, *NI-FAF* near-infrared fundus autofluorescence, *OD* right eye, *OS* left eye, *PRL* preferred retinal locus, *RPE* retinal pigment epithelium, *SD-OCT* spectral-domain optical coherence tomography, *SW-FAF* short-wavelength fundus autofluorescence. The median PRL eccentricity was 4.60° from the foveal centre (IQR 7.45°, range 0.09°–21.35°), median 63% BCEA was 3.5 deg^2^ (IQR 4.6 deg^2^, range 0.2 deg^2^–14.9 deg^2^), and median PRL sensitivity was 17.0 dB (IQR 9.0 dB, range 0.3–31.5 dB). Spearman’s correlation coefficients between participants’ right and left eyes were 0.90 (*p* < 0.001) for PRL eccentricity, 0.69 (*p* = 0.003) for 63% BCEA, 0.89 (*p* < 0.001) for retinal sensitivity, and 0.62 (*p* = 0.013) for PRL sensitivity.

The retinal structure at the PRL in participants with STGD1 was variable (Table 4). The RPE/Bruch’s membrane complex was present in all eyes except one where the PRL position on the retinal surface fell within an area of RPE atrophy. Autofluorescence signal (short-wavelength and near-infrared) was abnormal in most eyes and there was variable presence of the external limiting membrane and ellipsoid zone on SD-OCT. However, in areas of normal autofluorescence signal the external limiting membrane and ellipsoid zone were present.

### AOSLO images in Stargardt disease

Images were acquired over a range of retinal eccentricities (0°–20°). Cone photoreceptors were readily identifiable from the photoreceptor mosaic in areas of near normal retinal structure that had been identified on standard clinical images, however, in areas of retinal degeneration the photoreceptor mosaic was disorganised and lacked unambiguous cones. Hyper-reflective spots, termed discrete hyper-reflective foci, were identified in images from participants with Stargardt disease due to their relative hyper-reflectance compared to other image features. A continuous RPE cell mosaic was identified in one participant with an optical gap phenotype.

### Photoreceptor mosaic

**Fig. 1:**
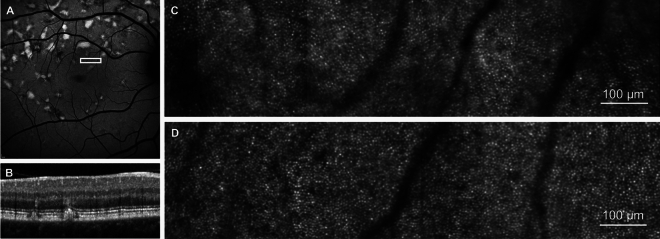
Photoreceptor mosaic in Stargardt disease in an area of near normal retina. (**A**) 30° short-wavelength fundus autofluorescence image, white box represents the retinal area corresponding to the photoreceptor mosaic in C and retinal cross section in B. (**B**) Spectral-domain optical coherence tomography of the retinal region represented by the white box in A. (**C**) Photoreceptor mosaic of participant S04. (**D**) Photoreceptor mosaic of an age-matched control (C05) to participant S04 from the same retinal eccentricity.

AOSLO images showed a regular photoreceptor mosaic in areas of normal or near normal retinal structure (Fig. 1). The retinal area corresponding to the photoreceptor mosaic illustrated in Fig. 1 demonstrates a relatively uniform SW-FAF signal with normal appearance of outer retinal layers on SD-OCT despite the evidence of adjacent retinal flecks.

**Fig. 2:**
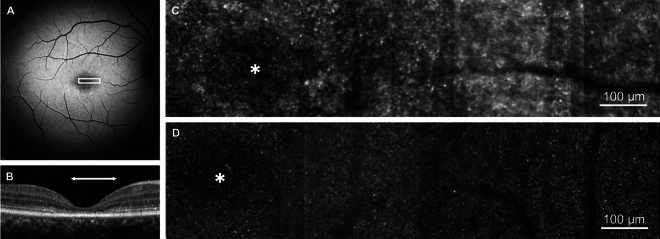
Photoreceptor mosaic in Stargardt disease in an area of degenerated retina. (**A**) 30° short-wavelength fundus autofluorescence image, white box represents the retinal area corresponding to the photoreceptor mosaic in C. (**B**) Spectral-domain optical coherence tomography. The horizontal white arrow indicates the retinal area that corresponds to the white box in A. (**C**) Photoreceptor mosaic of participant S06. (**D**) Photoreceptor mosaic of an age-matched control (C02) to participant S06 from the same retinal eccentricity. Asterisk represents the foveal centre.

In areas of retinal degeneration, the photoreceptor mosaic was disorganised and lacking unambiguous cones (Fig. 2). The retinal area corresponding to the photoreceptor mosaic in Fig. 2 demonstrates reduced SW-FAF signal and loss of the outer retinal layers on SD-OCT.

### Discrete hyper-reflective foci

**Fig. 3:**
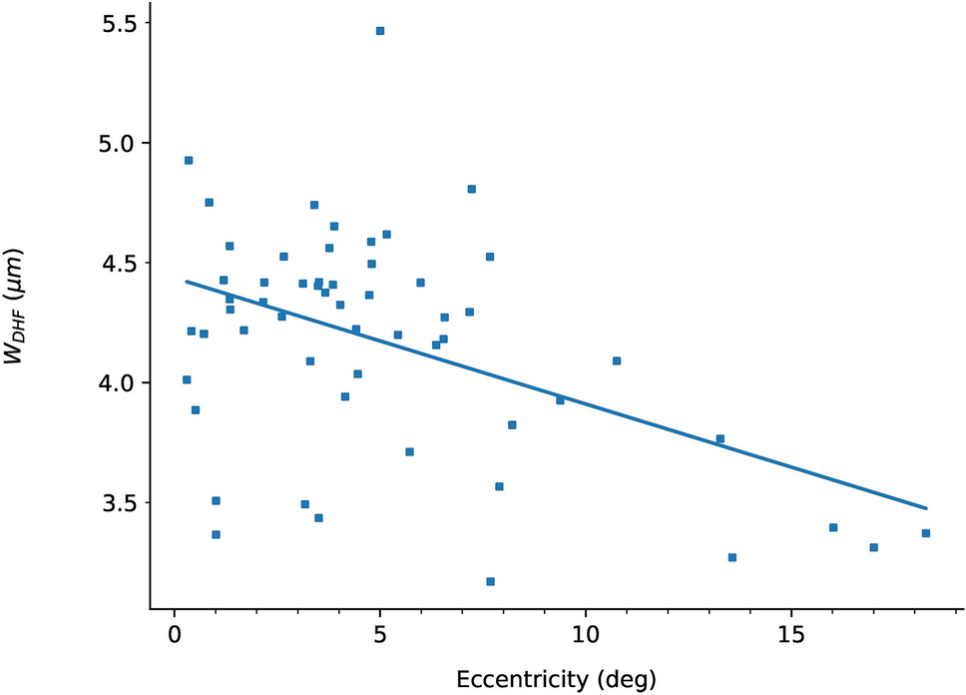
The relationship between retinal eccentricity and discrete hyper-reflective foci size. Each datum represents the size of an averaged image stack of 200 discrete hyper-reflective foci taken from a single AOSLO image. This is reported as full width at half maximum of a Gaussian profile fit to the average image. *W*_*DHF*_—full width at half maximum size of the discrete hyper-reflective foci in microns. The mean size of the discrete hyper-reflective foci for all participants and all retinal eccentricities is 4.1 μm (SD = 0.5 μm). The solid line represents a linear least-squares regression line of best fit (r = − 0.47, *p* < 0.001).

**Fig. 4:**
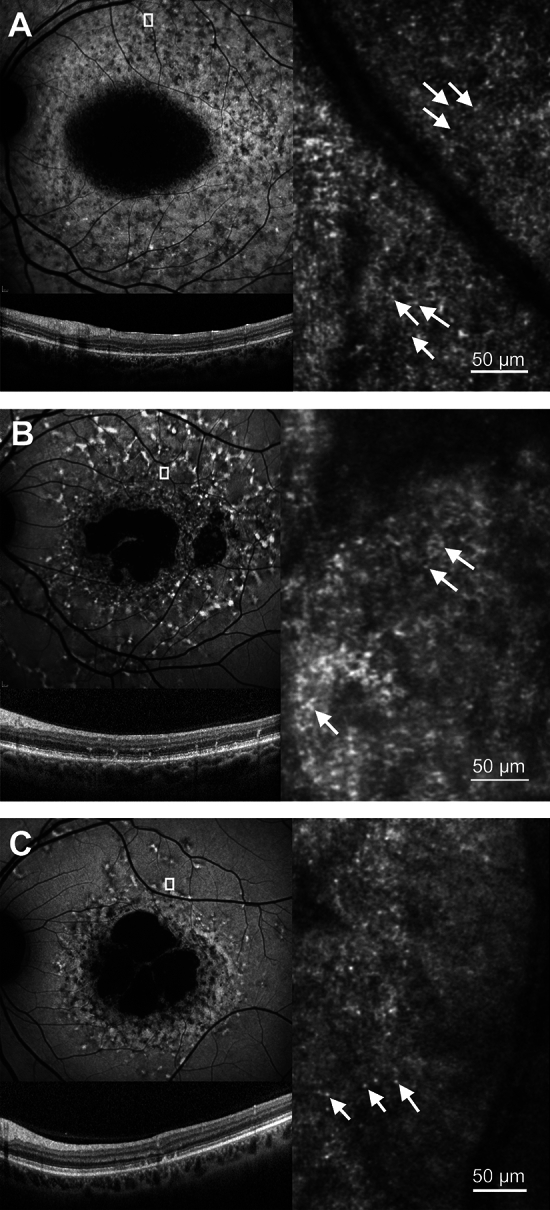
Examples of discrete hyper-reflective foci. 30° short-wavelength fundus autofluorescence (upper left), spectral-domain optical coherence tomography (lower left) and confocal AOSLO (right) images are shown. The white box in the short- wavelength fundus autofluorescence image represents the retinal area corresponding to the photoreceptor mosaic. White arrows indicate examples of the discrete hyper-reflective foci that can be seen throughout each image. (**A**) participant S11, (**B**) participant S14, (C) participant S15.

Discrete hyper-reflective foci were observed in nine participants with Stargardt disease, their size and distribution is illustrated in Fig. 3. A reduction in size of the discrete hyper-reflective foci was observed with increasing retinal eccentricity (r = − 0.47, *p* < 0.001). They were identified at retinal eccentricities ranging from 0.3° to 18.4° (Fig. 3) and by inspection lacked a regular packing arrangement. Examples of discrete hyper-reflective foci are shown in Fig. 4. The discrete hyper-reflective foci were only identified in areas with evidence of retinal degeneration on SD-OCT, autofluorescence and near-infrared reflectance imaging (Table 5).

**Table 5:** Discrete hyper-reflective foci in participants with Stargardt disease.

Participant	Eye	Eccentricity (degrees)		SW-FAF	NIR/NI-FAF	SD-OCT	Mean FWHM [SD] (µm)
		Min	Max				
S02	OD	0.3	6.0	Heterogeneous and hypo-AF	Heterogeneous NI reflectance	Disruption and/or loss of ELM and EZ	4.4 [0.2]
S03	OS	4.0	4.0	Heterogeneous AF	Heterogeneous NI reflectance	Loss of ELM and EZ	4.3 [0]
S05	OD	3.4	7.0	Heterogeneous AF	Reduced NI reflectance	Disruption and/or loss of ELM and EZ	4.4 [0.4]
S06	OD	0.5	1.0	Hypo-AF	Heterogeneous NI reflectance	Loss of ELM and EZ	3.6 [0.2]
S09	OS	0.3	6.2	Normal and hypo-AF	Heterogeneous NI reflectance in areas of normal SW-FAF	ONL thinning in areas of normal SW-FAF or hyper-reflective ELM and EZ loss	4.3 [0.1]
S11	OS	13.4	18.4	Heterogeneous AF	Heterogeneous AF	Loss of ELM and EZ	3.4 [0.2]
S12	OS	5.9	8.0	Hypo-AF	Hypo-AF	Hyper-reflective foci within ONL	3.5 [0.2]
S14	OS	7.6	7.6	Heterogeneous AF	Heterogeneous AF	Disruption of ELM and EZ	4.3 [0]
S15	OS	10.5	10.9	Heterogeneous AF	Hypo-AF	Disruption of ELM and EZ	4.0 [0.1]

*NIR* near infrared reflectance imaging. *NI-FAF* near-infrared fundus autofluorescence, *OD* right eye, *OS* left eye, *SD-OCT* spectral-domain optical coherence tomography, *SW-FAF* short-wavelength fundus autofluorescence.

### Retinal pigment epithelium mosaic

**Fig. 5:**
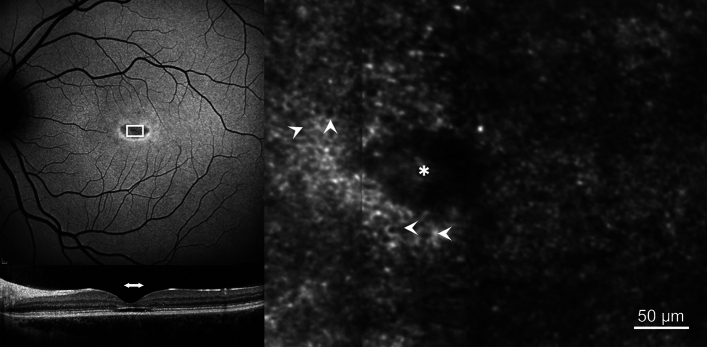
Retinal pigment epithelial (RPE) mosaic. 30° short-wavelength fundus autofluorescence (SW-FAF, upper left), spectral-domain optical coherence tomography (SD-OCT, lower left) and confocal AOSLO (right) images are shown. The white box in the SW-FAF image represents the retinal area corresponding to the photoreceptor mosaic. The horizontal white arrow in the SD-OCT image indicates the retinal area that corresponds to the white box in the SW-FAF image. The white arrowheads in the confocal AOSLO image indicate examples of the RPE cells visible throughout the image. Asterisk represents the foveal centre.

The characteristic appearance of a continuous RPE cell mosaic (hexagonal cells with a dark centre surrounded by a brighter ring made up of discrete spots) was clearly observed in one participant (S09) with an optical gap phenotype^12^ (Fig. 5). No clearly discernible photoreceptors were identifiable within the area of focal ellipsoid zone loss. The area of foveal cavitation was characterised by reduced SW-FAF signal with focal loss of the outer nuclear layer and ellipsoid zone on SD-OCT. Despite the evidence of retinal degeneration and absence of clearly discernible photoreceptors, best corrected visual acuity was 0.08 LogMAR with a foveal PRL (0.03°) and mean PRL sensitivity of 16.0 dB (SD 4.2 dB) (Tables 1 and Table 4).

## Discussion

This study demonstrates that the clinical heterogeneity observed in STGD1 is reflected in the findings on confocal AOSLO imaging. A regular photoreceptor mosaic was observed in areas of near normal retinal structure on clinical images, but in areas of retinal degeneration it was disorganised with a lack of unambiguous cones. Structural changes were observed using confocal AOSLO imaging that are not detectable with conventional clinical imaging modalities. The RPE mosaic appeared qualitatively regular in its packing arrangement and discrete hyper-reflective foci were observed throughout areas of retinal degeneration.

There are a limited number of publications reporting the results of AOSLO imaging in patients with STGD1^13–28^. Investigations of early STGD1 with in vivo AOSLO imaging by Song et al*.*^13^ demonstrated evidence of centrifugal disease progression from the fovea. They observed areas of increased rod and cone photoreceptor spacing in areas of normal conventional clinical imaging, suggesting that photoreceptor loss precedes clinically detectable RPE disease^13^.

The regular photoreceptor mosaics observed in this study in areas of normal or near normal retinal structure on clinical images were qualitatively similar to those in controls. They showed regular packing and the spatial density was comparable. Cone-counting based on human coders or automated methods is imperfect, and susceptible to factors affecting image quality, such as the changes in fixation stability seen in patients with STGD1 that result in image motion artefacts. In areas of retinal degeneration the identification of unambiguous cones is particularly challenging, which makes cone-counting difficult. We therefore chose not to perform formal analyses of cone counts in this study but rely on qualitative descriptions.

Longitudinal studies of AOSLO imaging in patients with Stargardt disease have reported that the earliest cone photoreceptor spacing abnormalities occur in areas of homogeneous SW-FAF, normal vision and normal outer retinal structure on SD-OCT^16^. These changes are followed by a heterogeneous increase in SW-FAF signal with cone loss, and then by a reduction of SW-FAF signal with cone and RPE cell death^16^. In vivo autofluorescence AOSLO imaging in patients with Stargardt disease has demonstrated patterns of autofluorescence that appear to colocalise with photoreceptors^14^. These observations support histologic reports of photoreceptor bisretinoid accumulation^29^ and may be useful as an early biomarker in patients with Stargardt disease. New methods to facilitate the interpretation of complex multimodal imaging datasets from patients with Stargardt disease with comparison of structural and functional information^15^, as well as methods for the identification of cone photoreceptors^30–32^ have also been described.

### Discrete hyper-reflective foci

Discrete hyper-reflective foci observed on AOSLO imaging in this study were identified in areas of disruption or loss of the external limiting membrane and ellipsoid zone on SD-OCT and abnormal autofluorescence and near-infrared reflectance signals. Due to their small size they were not visible on SD-OCT. The observation of hyper-reflective structures has been described in AOSLO images from patients with diabetes^33,34^ and central serous chorioretinopathy^35^. They have also been previously reported in Stargardt disease corresponding to cone photoreceptors within the central rod free zone^36^ and rod photoreceptors at more peripheral retinal eccentricities^28^. Hyper-reflective structures have also been observed on SD-OCT in patients with STDG1 across the retina and choroid where they have been reported to correlate with disease severity^37,38^. Suggested hypotheses regarding the aetiology of these hyper-reflective foci include aggregates of activated microglial cells^39^, degenerated photoreceptors^40^, and migrating RPE cells^41^. The small size of the hyper-reflective foci observed in this study makes it unlikely that they are migrating RPE cells (diameter 10–14 μm^42,43^) or activated macrophages (diameter 21 μm^44^) and without histological confirmation it is not possible to definitively determine if they represent microglial cells in a degenerating retina. The discrete hyper-reflective foci demonstrate an eccentricity dependent reduction in size (Fig. 3), which is consistent with a shift in the balance of (larger) cones to (smaller) rods that is expected from previously published data on increasing rod photoreceptor density with eccentricity^45^. Thus the discrete hyper-reflective foci in this study may represent cone or rod photoreceptors depending on the eccentricity. However, with confocal imaging the size of the structure being imaged and its reflectivity can be misleading such that the size of more reflective structures can be overestimated. Therefore, cone and rod photoreceptor outer segments at the same eccentricity can appear to be similar in confocal imaging. Without repeating these images using non-confocal AOSLO imaging (which is useful in identifying photoreceptors from preserved inner segments), it is not possible to be certain of their origin.

### *ABCA4*-associated optical gap phenotype

The optical gap phenotype associated with Stargardt disease is characterised by foveal cavitation that appears to represent a focal loss of ellipsoid zone reflectance on OCT^12^. In this study there were no clearly discernible cone photoreceptors observed within the fovea of a participant with an optical gap phenotype. Loss of the ellipsoid zone reflectance (which represents the photoreceptor inner segment/outer segment junction) on SD-OCT is consistent with the lack of unambiguous cones on AOSLO imaging, which represents reflections from the cone inner segment/outer segment junction and outer segment/RPE interface. Despite the lack of cone photoreceptors, there was evidence of visual function. An incongruous relationship between the extent of retinal degeneration and visual acuity has previously been reported in patients with an *ABCA4*-associated optical gap phenotype, which has been attributed to preservation of photoreceptors at the PRL^12^.

In this study the presence of foveal cavitation on SD-OCT with loss of ellipsoid zone and the observation of a continuous RPE mosaic within the fovea (which is easier to image in AOSLO without photoreceptors) suggest either a loss of photoreceptors or a change to their wave-guiding properties caused by degeneration. Some degenerated photoreceptors may remain intact in participants with an optical gap phenotype and in such cases, the addition of complementary non-confocal imaging modalities may be useful in identifying preserved photoreceptors. In participant S09 the bivariate contour ellipse area suggests poorer fixation stability and preserved ellipsoid zone on SD-OCT immediately surrounding the fovea implies preservation of photoreceptors and functional retina. These suggest an eccentric PRL within the functional retina around the fovea as a more likely explanation of the maintained central visual function in this participant with an *ABCA4*-associated optical gap phenotype.

### Retinal pigment epithelium mosaic

The signal from photoreceptors in confocal AOSLO imaging is so strong that to see other structures, such as RPE cells, it needs to be blocked by using non-confocal imaging geometries. Dark-field reflectance imaging with the AOSLO is typically required to directly visualise individual RPE cells, but a continuous RPE mosaic was observed within the fovea in this study. To date, there has been one report describing the RPE cell mosaic in patients with STGD1 using AO in vivo autofluorescence imaging^14^. The RPE mosaic described by Song et al*.* was visualised in the retinal periphery in areas with normal photoreceptors. In more central areas with disrupted cone and rod photoreceptors they described abnormal autofluorescent structures that appeared to be more consistent with photoreceptor reflectance rather than RPE cells^14^.

Limitations of this study include the lack of non-confocal AOSLO imaging, a small sample size, and the cross-sectional nature of the data. Thus this cohort cannot be fully representative of a condition that is both clinically and genetically highly heterogeneous. Larger studies with multimodal imaging will be required to validate the findings from this cohort.

## Conclusions

This study adds to the literature by reporting AOSLO imaging features in a heterogenous cohort of participants with STGD1 and highlights the potential for AOSLO to become a useful tool to monitor the retina in health and disease. Future studies that incorporate high-resolution retinal imaging using alternative imaging modalities such as split-detection, non-confocal (dark-field) reflectance and autofluorescence, coupled with functional cellular information will aid in the identification and interpretation of relevant image features.

## Supplementary Information

Supplementary Figure 1.

## Data Availability

The datasets generated and/or analysed during the current study are not publicly available due patient confidentiality but are available from the corresponding author on reasonable request.
