# Supplementary material for: Adaptive optics scanning laser ophthalmoscopy in a heterogenous cohort with Stargardt disease
Source: Sci Rep. 2024 Oct 9;14:23629. doi: 10.1038/s41598-024-74088-y (PMC11464663; doi:10.1038/s41598-024-74088-y)
Supplement: Supplementary file 1 — Supplementary Figure 1. [file 41598_2024_74088_MOESM1_ESM.docx]

**
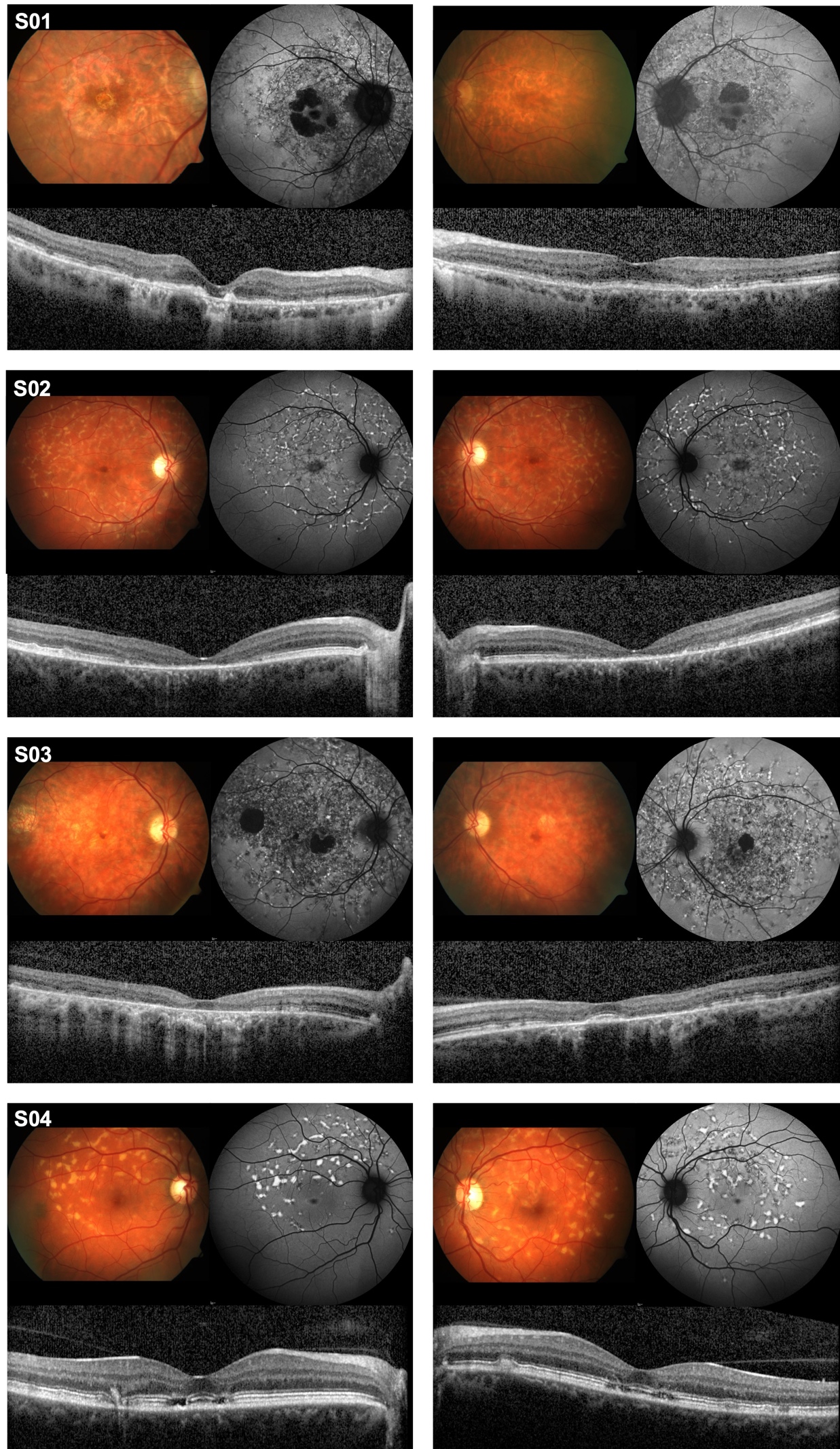
**

**
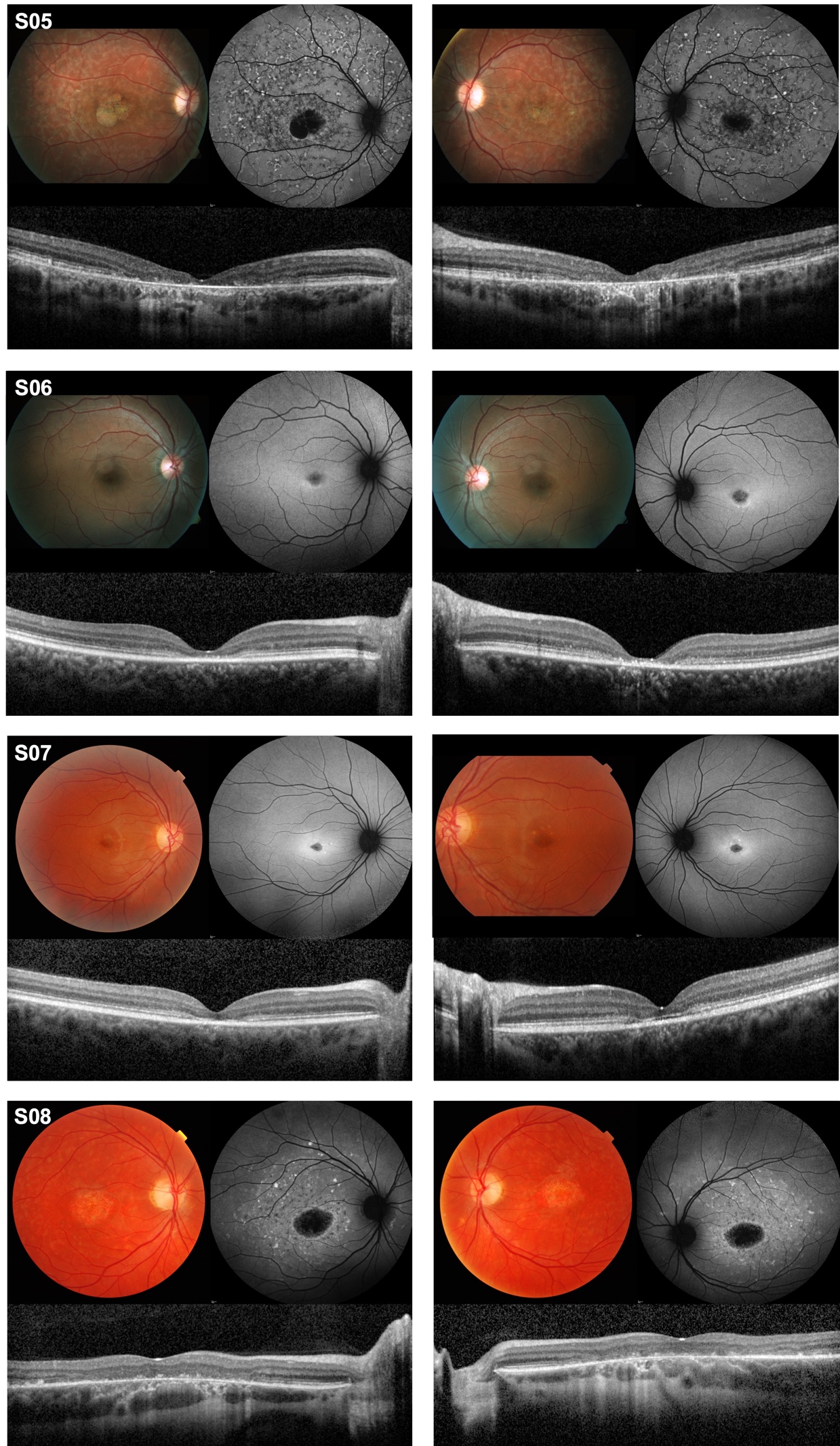
**

**
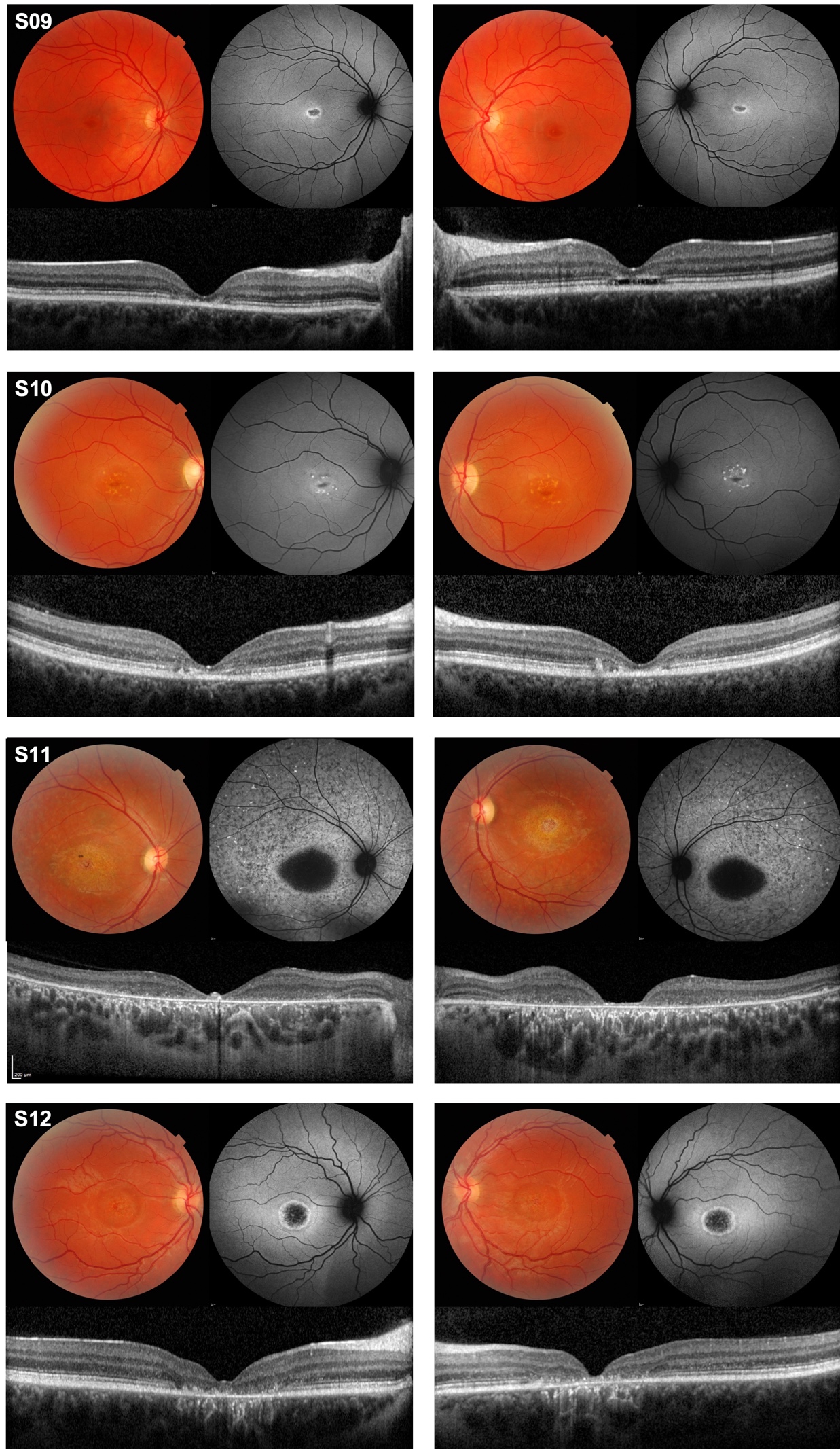
**

**
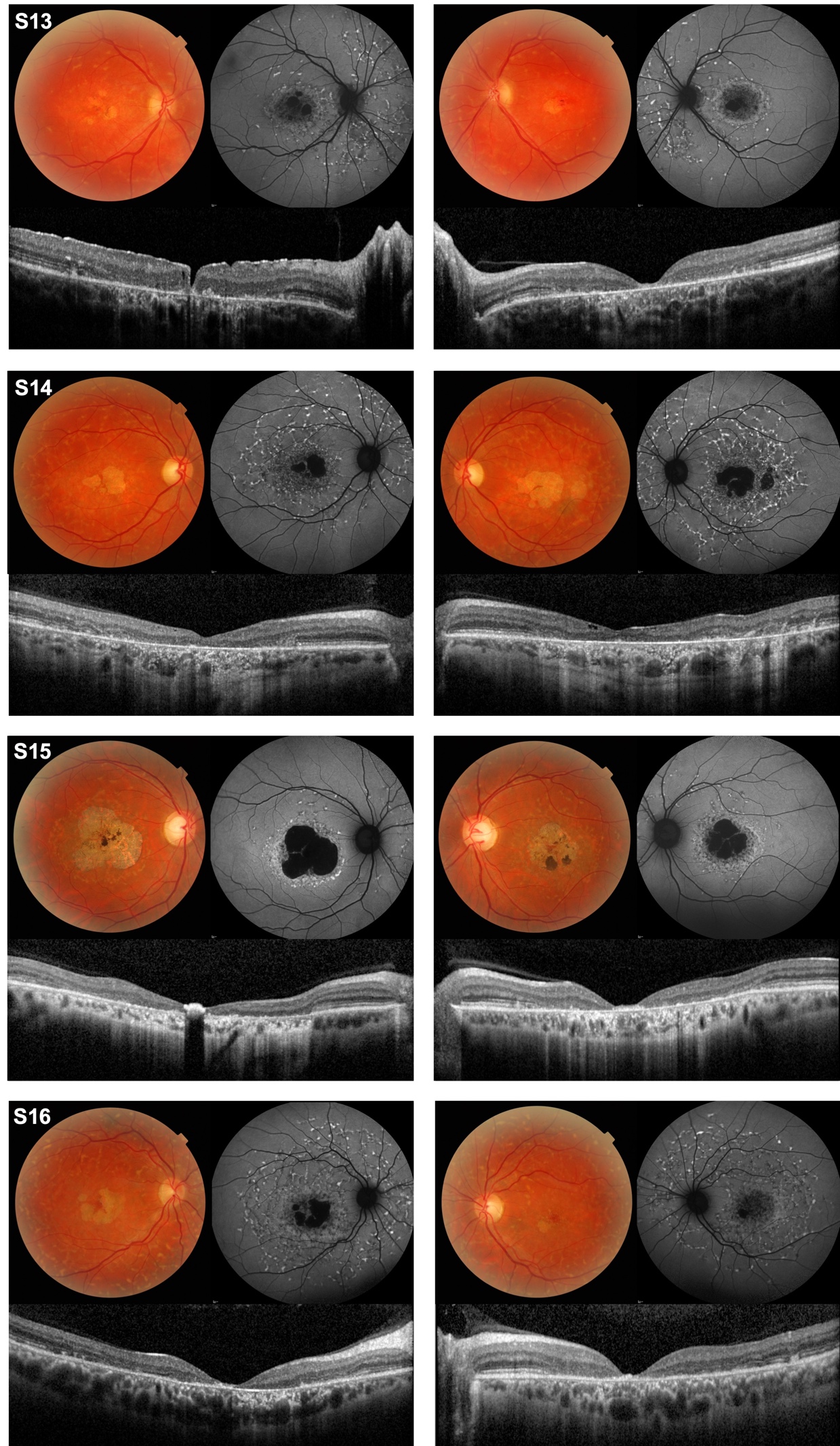
**

**Supplementary Figure 1 - Retinal phenotype of participants with Stargardt disease.** For each participant the panels show: 55° short-wavelength fundus autofluorescence (top left); fundus colour (top right); and spectral-domain optical coherence tomography providing a cross-section through the fovea (bottom). Images of both eyes (left column - right eyes; right column - left eyes) are shown.
